# Complex Processing Patterns of mRNAs of the Large ATP Synthase Operon in *Arabidopsis* Chloroplasts

**DOI:** 10.1371/journal.pone.0078265

**Published:** 2013-11-04

**Authors:** Mustafa Malik Ghulam, Florence Courtois, Silva Lerbs-Mache, Livia Merendino

**Affiliations:** 1 Laboratoire de Physiologie Cellulaire & Végétale, UMR 5168, CNRS, Grenoble, France; 2 Laboratoire de Physiologie Cellulaire & Végétale, Univ. Grenoble Alpes, Grenoble, France; 3 Laboratoire de Physiologie Cellulaire & Végétale, CEA, DSV, iRTSV, Grenoble, France; 4 Laboratoire de Physiologie Cellulaire & Végétale, USC1359, INRA, Grenoble, France; National Taiwan University, Taiwan

## Abstract

Chloroplasts are photosynthetic cell organelles which have evolved from endosymbiosis of the cyanobacterial ancestor. In chloroplasts, genes are still organized into transcriptional units as in bacteria but the corresponding poly-cistronic mRNAs undergo complex processing events, including inter-genic cleavage and 5′ and 3′ end-definition. The current model for processing proposes that the 3′ end of the upstream cistron transcripts and the 5′ end of the downstream cistron transcripts are defined by the same RNA-binding protein and overlap at the level of the protein-binding site.

We have investigated the processing mechanisms that operate within the large ATP synthase (*atp*) operon, in *Arabidopsis thaliana* chloroplasts. This operon is transcribed by the plastid-encoded RNA polymerase starting from two promoters, which are upstream and within the operon, respectively, and harbors four potential sites for RNA-binding proteins. In order to study the functional significance of the promoters and the protein-binding sites for the maturation processes, we have performed a detailed mapping of the *atp* transcript ends. Our data indicate that in contrast to maize, *atpI* and *atpH* transcripts with overlapping ends are very rare in *Arabidopsis*. In addition, *atpA* mRNAs, which overlap with *atpF* mRNAs, are even truncated at the 3′ end, thus representing degradation products. We observe, instead, that the 5′ ends of nascent poly-cistronic *atp* transcripts are defined at the first protein-binding site which follows either one of the two transcription initiation sites, while the 3′ ends are defined at the subsequent protein-binding sites or at hairpin structures that are encountered by the progressing RNA polymerase. We conclude that the overlapping mechanisms of mRNA protection have only a limited role in obtaining stable processed *atp* mRNAs in *Arabidopsis*. Our findings suggest that during evolution of different plant species as maize and *Arabidopsis*, chloroplasts have evolved multiple strategies to produce mature transcripts suitable for translation.

## Introduction

Chloroplasts are cell organelles in photosynthetic organisms where essential functions as photosynthesis, synthesis of lipids, pigments, vitamins and amino acids occur. They have evolved from endosymbiosis of photosynthetic cyanobacteria and from those they have inherited many prokaryotic-like elements. However, during the evolution process from bacteria, chloroplasts have acquired also eukaryotic features and plastid-specific characteristics [1]. The gene-expression system represents a good example for the acquirement of complexity in plastids. In chloroplasts, genes are still organized into transcriptional units or operons as in bacteria. However, differently from the bacterial counterparts, the corresponding poly-cistronic mRNAs undergo complex processing events and only upon intron splicing, editing, inter-cistronic cleavage and *termini* definition, the mature mRNAs are ready for translation.

An early model for inter-cistronic processing of chloroplast mRNAs predicts that endonucleases cut at specific sites and exonucleases then trim the RNA until an hairpin is encountered [2]. More recently, pentatricopeptide repeat (PPR) proteins together with other classes of RNA-binding proteins have been involved in the definition of transcript *termini*. Small RNAs (sRNAs) persisting in the RNA pool even upon mRNA degradation have been identified as footprints of RNA-binding proteins [3], [4]. The finding of transcripts of adjacent cistrons having overlapping ends was explained by a novel mechanism in which the 3′ end of the upstream cistron transcripts and the 5′ end of the downstream cistron transcripts are protected from exonucleolytic degradation by the same RNA-binding protein [5]. PPR10 was shown to protect the overlapping ends of *atpI* and *atpH* mRNAs in maize chloroplasts [6].

Recently, we have analyzed the transcriptional organization of the plastid large ATP synthase (*atp*) operon of *Arabidopsis thaliana* that consists of the genes *atpI/H/F/A* [7]. This operon is transcribed by the plastid-encoded RNA polymerase (PEP) starting from a promoter at −225/229 in front to the *atpI* coding sequence, which is dependent on the Sigma factor 2 (SIG2). In addition, we identified an operon-internal promoter just upstream of the *atpH* ORF, which is dependent on the Sigma factor 3 (SIG3) [8]. This *atpH* specific promoter has not been described in maize thus raising the question of whether the processing mechanisms of the *atp* operon mRNAs are comparable in maize and *Arabidopsis*. Furthermore, in addition to the supposed PPR10-binding site in the *atpI/H* inter-genic region, three other sites for RNA-binding proteins were predicted in the large *atp* operon, upstream of the *atpI* ORF and within the *atpH/F* and the *atpF/A* inter-genic regions [3], [4]. In order to elucidate the mechanisms controlling the processing of the *atpI/H/F/A* transcripts in *Arabidopsis* chloroplasts as well as the functional significance of the operon-internal promoter and the four protein-binding sites for these maturation processes, we have performed a detailed mapping of the 5′ and 3′ ends of the complete set of mRNAs encoded by the large *atp* operon. Our data show that 5′ ends of the nascent poly-cistronic *atp* transcripts are defined at the first protein-binding site following the transcription initiation sites, while the 3′ ends are defined at the subsequent protein-binding sites or hairpin structures that are encountered by the progressing RNA polymerase. We observe that *atp* transcripts with overlapping ends are rare and in some cases they are even truncated at the 3′ end, thus representing degradation products.

## Results

### Mapping strategy

**Figure 1:**
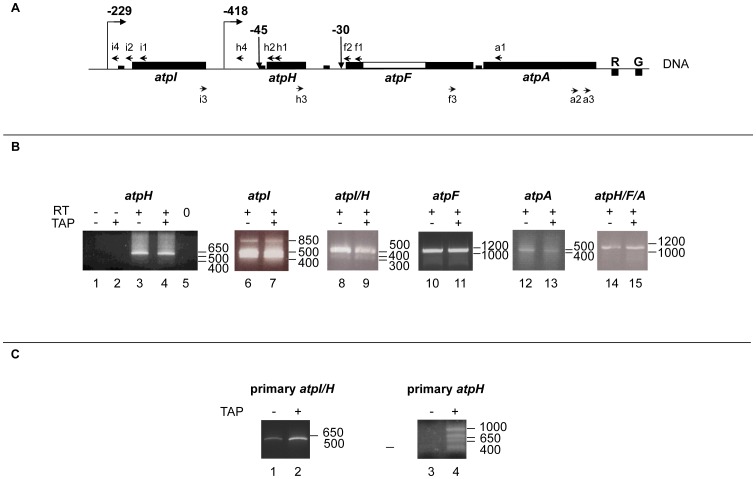
Mapping of transcript ends generated from the large *atp* operon in *Arabidopsis* chloroplasts. A. Schematic presentation of the *atpI/H/F/A* operon structure. Filled thick boxes correspond to coding sequences of the *atp* and *trn* (R and G) genes that are present on opposite DNA strands. The empty box corresponds to the *atpF* intron. The small filled rectangles correspond to the sRNAs [3], [4]. Upward (with right tip) and downward arrows indicate the 5′ end positions of primary and processed transcripts, respectively, with negative numbers corresponding to the distance from the ATG as described [7]. Left and right-directed arrow heads correspond to primers for the cRT-PCR analysis. B. Mapping of processed *atp* transcript ends by cRT-PCR. Agarose gels showing the cRT-PCR data are presented, with the positions of the molecular weight markers on the right. For *atpH* transcripts (lanes 1–5), h1 was used as RT-primer and h2–h3 as PCR-primers. For *atpI* transcripts (lanes 6–7), i1 was used as RT-primer and i2–i3 as PCR-primers. For *atpI/H* transcripts (lanes 8–9), i1 was used as RT-primer and i2–h3 as PCR-primers. For *atpF* transcripts (lanes 10–11), f1 was used as RT-primer and f2–f3 as PCR-primers. For *atpA* transcripts (lanes 12–13), a1 was used as RT-primer and a1–a3 as PCR-primers. For *atpH/F/A* transcripts (lanes 14–15), h1 was used as RT-primer and h2–a2 as PCR-primers. 5′ processed transcripts were distinguished from primary mRNAs because PCR products were also obtained in absence (-TAP) of a previous TAP-treatment of the RNA samples. The -RT (lanes 1 and 2) and -PCR (lane 5) controls are shown only for the mono-cistronic *atpH* mRNAs. C. Mapping of primary *atp* transcript ends by cRT-PCR. For *atpI/H* transcripts (lanes 1–2), i1 was used as RT-primer and i4–h3 as PCR-primers. For *atpH* transcripts (lanes 3–4), h1 was used as RT-primer and h4–h3 as PCR-primers.

**Figure 2:**
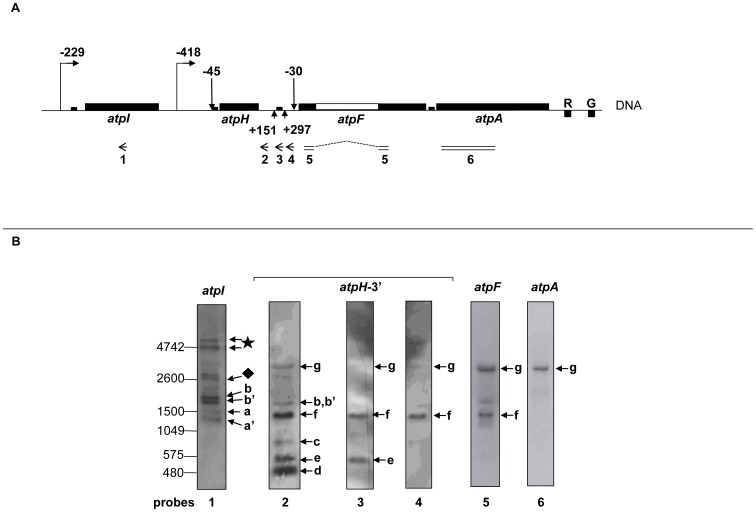
Northern blot analyses of transcripts generated from the large *atp* operon in *Arabidopsis* chloroplasts. A. Schematic presentation of the *atpI/H/F/A* operon structure is as described in Figure 1A. Upward arrows indicate the position of 3′ ends of the *atpH* transcripts from the corresponding stop codon (+151 and +297), on the bases of the circular RT-PCR data obtained in this manuscript. Left arrows (1 to 4) and double lines (5 and 6) correspond to single and double strand DNA probes, respectively, which are used in the Northern blot analysis. The probe 5 was designed to detect only the spliced form of *atpF* transcripts. B. Northern blot analyses of the *atp* transcripts. The analysis was performed on total RNA using *atp* gene-specific probes (1 to 6). The multiple transcript isoforms are indicated with letters (corresponding to transcripts in Figure 3A) or symbols (diamond, star). The positions of the molecular weight markers are indicated on the left of the agarose gels.

For mapping of transcript ends, we have chosen the circular RT-PCR (cRT-PCR) technique because this method allows determination of the 5′ and 3′ ends delimiting the same RNA molecule [9]. Conversely, techniques like 5′ and 3′ RACE, primer extension or nuclease S1 mapping allow definition of only one of the two mRNA ends and cannot distinguish among different combinations of 5′ and 3′ ends. We isolated total RNA from 7 day old *Arabidopsis* seedlings. Transcripts were self-ligated and retro-transcribed with gene-specific primers for the single cistrons in the *atp* operon (Figure 1A). cDNA fragments containing the fused 5′ and 3′ ends were then amplified for cloning (when necessary) and sequencing (Figure 1B). In order to characterize also primary transcripts, RNA self-ligation was preceded by treatment with Tobacco acid pyro-phosphatase (TAP) (Figure 1C). Data obtained by cRT-PCR were then complemented by Northern blot analyses, which demonstrate the relative ratio among the different RNA populations (Figure 2).

### Mapping of the mono-cistronic *atpH* transcript ends

**Figure 3:**
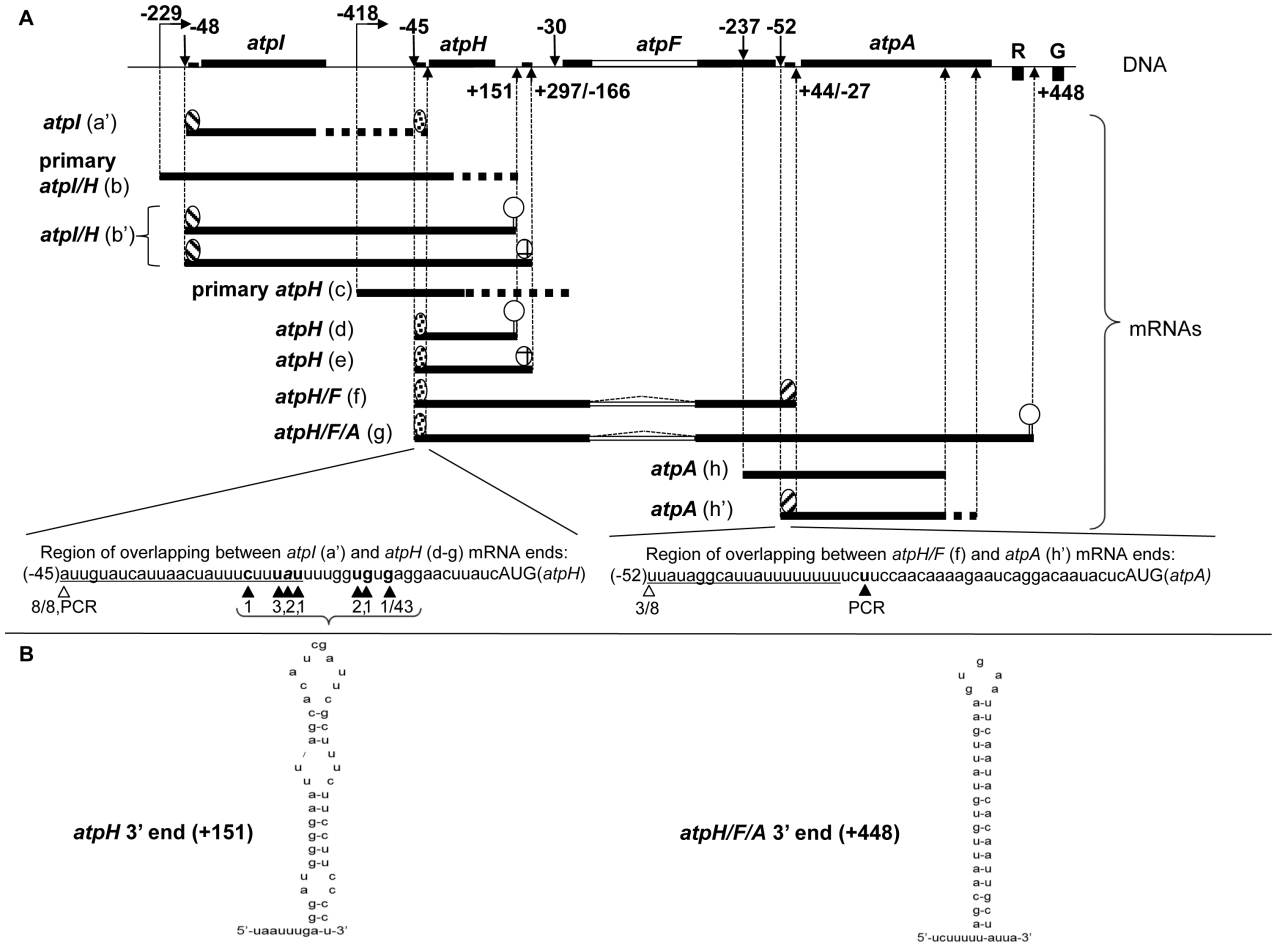
Schematic presentation of the *atpI/H/F/A* operon and the encoded transcripts. A. Top panel: the schematic presentation of the *atpI/H/F/A* operon is as in Figures 1A and 2A. Downward and upward arrows indicate the position of 5′ and 3′ ends of the *atp* transcripts, respectively, on the bases of cRT-PCR (this manuscript) and primer-extension data [7]. For the 3′ ends of the *atpI/H* (b′), *atpH* (e) and *atpH/F* (f) mRNAs, both the distance from the corresponding stop codon and from the start codon of the downstream cistrons is indicated. Middle panel: transcripts are labeled by letters as in Figure 2B. The thick continuous lines correspond to *atp* transcripts containing defined ends; the discontinuous lines of the (a′, b, c and h′) *atp* mRNAs indicate the presence of dispersed 3′ ends. Distinct RNA-binding proteins that are likely involved in definition of transcript ends are depicted as circles with different fillings. 3′ ends that are likely protected by stem-loop structures are depicted as stem loops. The triangle (dotted line) within the *atpF* mRNA indicate splicing of the intron. Bottom panel: the sequence of overlapping regions between *atp* transcript ends is indicated. The numbers below empty or full arrowheads correspond to the ratio of the clones displaying a specific 5′ or 3′ transcript end, respectively. The term “PCR” indicates when sequencing was performed directly on the PCR product. The sequence corresponding to the sRNAs, as reported in [3], [4] is underlined. B. Structure prediction of *atp* mRNA 3′ ends lacking sRNAs. Linear RNA folding at 25°C of the last 50 nucleotides of the *atpH* transcripts with a 3′ end at position +151 (left panel, ΔG = −14.36 kcal/mol) and of the *atpH/F/A* transcripts with a 3′ end at position +448 (right panel, ΔG = −29.49 kcal/mol).

We started our analysis by mapping the ends of the *atpH* transcripts, which are the most abundant mRNAs encoded by the large *atp* operon [10]. When primers were designed inside the coding region of *atpH* mRNAs (h2 and h3 primers, Figure 1A), a sharp PCR product was obtained in both TAP-untreated and treated retro-transcribed RNAs (Figure 1B, lanes 3 and 4), indicating that the transcripts were generated by a processing event at the 5′ end. In parallel, amplification reactions without previous reverse transcription (lanes 1–2) or DNA template (lane 5) were carried out as controls for the specificity of the cRT-PCR reaction. The PCR product was then cloned and 8 clones were sequenced. All sequences indicated 5′ ends at position −45 (Figure 3A, bottom panel) and most of the clones (6/8) 3′ ended in the inter-genic region between *atpH* and *atpF*, at position +297 from *atpH* stop codon (e in Figure 3A). A short RNA (sRNA) with a 5′ end at position −45 was also found in *Arabidopsis* [3] and in maize [4] chloroplasts. This sRNA corresponds to the PPR10-binding site in maize. Another sRNA with a 3′ end around position +297 was also discovered in *Arabidopsis* chloroplasts [3]. Taken together, these data suggest that mono-cistronic *atpH* mRNAs starting at position −45 and ending at position +297 are generated through protection by RNA-binding proteins. However, we detected also another mono-cistronic *atpH* isoform with a 3′ end at position +151 (d in Figure 3A), a site that was already found using the 3′ RACE technique. No sRNAs were found to map in close vicinity to this site [3]. We performed a secondary structure prediction using the MFOLD 3.2 program (http://mobyle.pasteur.fr/cgi-bin/portal.py?#forms), which indicated that the 50 nucleotides upstream to the +151 site can fold into a stable stem-loop structure with a predicted ΔG of −14.36 kcal/mol (Figure 3B, left panel). Similar structures were proposed to protect the ends of plastid mRNAs as an alternative to the protein-mediated mechanism [2], [5]. Interestingly, we found that the *atpH* transcript 3′ end at the +151 site included an extra sequence (AA), suggesting that this mRNA represents an intermediate product of a processing/degradation event [11]. These data indicate that the *atpH* transcripts are mostly present as two distinct mono-cistronic isoforms which share the same 5′ processed end nearby the PPR10-binding site. However, the longer isoform (e in Figure 3A) contains a well-defined 3′ end which maps close to a protein footprint, while the shorter one (d in Figure 3A) presents a 3′ end which maps close to a stem loop structure. The existence of these two types of transcripts was confirmed with Northern blot analyses (d and e in Figure 2B) that were performed with single strand DNA probes hybridizing to the *atpH* coding region (data not shown) or to the 3′UTR (Figure 2A, probe 2–4). When probe 2 was used, which hybridizes to the *atpH* 3′ UTR just upstream to the +151 site, three very abundant bands (d, e and f) were revealed (Figure 2B). The bands e and f, but not the band d, were still detected when we used probe 3 which was designed to hybridize in between the hairpin structure (+151) and the protein-binding site (+297). Finally, only the band f was detected when we used probe 4 which was hybridizing directly downstream to the protein-binding site (+297). Thus, in addition to the confirmation of two different mono-cistronic *atpH* transcripts, the Northern experiment revealed the existence of a longer *atpH* transcript (band f), that corresponds to an *atpH/F* co-transcript (see below).

### Mapping of the *atpI* transcript ends

Genetic and biochemical data have shown that the same barrier protein, PPR10, is involved in the stabilization and definition of the overlapping ends of the *atpH* and *atpI* mRNAs in maize [6]. However, the transcriptional organization of the large *atp* operon differs in *Arabidopsis* and in maize with respect to the internal *atpH* promoter [8]. Therefore, processing mechanisms might be also different in these two plant species. For that, we have verified the validity of the overlapping model at this site in *Arabidopsis* by mapping the ends of the *atpI* transcripts. Amplification of circularized *atpI* mRNAs using the i2 and i3 primers (Figure 1A) revealed a diffuse band pattern with both TAP-untreated and treated RNA samples (Figure 1B, compare lanes 6 and 7), where all *atpI* mRNAs result from a 5′ processing event. We have sequenced 11 clones and found that 9 sequences revealed mRNA 5′ ends at position −48 from *atpI* AUG whereas 3′ ends were scattered all over the *atpI/H* inter-genic region (a′ in Figure 3A). One of the clones (1/11) presented an extra sequence at the 3′ end, indicating it as an intermediate of a RNA processing/degradation event. Only 2 clones (2/11) displayed 3′ ends just downstream to the PPR10 sRNA mapping site (this mRNA is expected to be 1292 nucleotides long) (Figure 3A, bottom panel). We have repeated this experiment twice, using two independent preparations of RNAs that were obtained from two independent sets of plants. All the clones analyzed (32 clones) revealed mRNA 5′ ends at position −48, indicating a very stable 5′ end. Only a limited proportion of the clones (9/32) revealed 3′ ends inside or just downstream to the PPR10 footprint (Figure 3A, bottom panel). In addition, 12/32 and 7/32 clones displayed 3′ ends at positions +5 and +390 downstream to the *atpI* stop codon, respectively, indicating two additional 3′ ends. These mRNAs are expected to be 803 and 1188 nucleotides long, respectively. As in the precedent experiment, few (3/32) clones presented extra sequences. Our data indicate that even though in *Arabidopsis* the *atpH* 5′ end is well protected against nuclease digestion (likely by the PPR10 orthologue) as in maize, the 3′ end of the *atpI* mono-cistronic transcripts is not defined with the same efficiency. This suggests that the mechanism for the *atpI/H* transcript processing in *Arabidopsis* chloroplasts is different from that in maize. On the other hand, when the reverse (i2) and forward (h3) primers were designed inside the *atpI* and the *atpH* coding sequences, respectively, in order to characterize the longer *atpI/H* co-transcripts (see Figure 1A), two sharp PCR products were amplified after reverse transcription of circularized mRNAs (Figure 1B, lanes 8 and 9). 9 clones were analyzed and all mRNAs displayed 5′ ends at position −48 from the *atpI* AUG and 3′ ends either at position +151 (2 of 9 clones) or at position +295 (5 of 9 clones) downstream to the *atpH* stop codon (b′ in Figure 3A). These data demonstrate that stable *atpI* mRNAs do exist mainly as di-cistronic *atpI/H* mRNAs, which use the same barrier modules for 3′ end definition of the *atpH* mono-cistronic mRNAs.

As *atpI* mRNA could also result from co-transcription with the preceding *rps2* gene, we performed Northern blot hybridizations using single strand DNA probes complementary to either *rps2* (data not shown) or *atpI* (Figure 2A and B, probe 1) coding regions. Only one mRNA of about 2600 nucleotides (diamond) was revealed by both probes, suggesting that the remaining *atpI* bands correspond to transcripts which initiate at the *atpI* cistron. Two bands (a′ and a) of around 1300 and 1500 nucleotides, respectively, were detected, that from the size could correspond to *atpI* mono-cistronic mRNAs. In our previous analyses we have observed that only the larger of the two bands (a) disappeared in the *sig2* mutant [7]. These data suggested that the larger and very faint band (a) corresponds to the SIG2 dependent primary *atpI* transcripts (starting at position −229) while the smaller band (a′) corresponds to the processed *atpI* mRNAs (with the 5′ end at position −48, as based on our cRT-PCR data). Two bands of the expected size for di-cistronic *atpI/H* transcripts (around 1800 nucleotides) were detected (bands b and b′). They were also revealed by DNA probes hybridizing to the *atpH* coding region (data not shown) or the 3′ UTR (Figure 2B, probe 2). Our previous analyses have shown that only the larger of these two bands (b) was SIG2 dependent, corresponding to the SIG2-dependent primary *atpI/H* co-transcripts (starting at position −229, see below), while the smaller band (b′) likely corresponds to the processed *atpI/H* mRNAs (with the 5′ end at position −48 on the basis of the cRT-PCR data in this manuscript) [7].

### Mapping of the *atpF* and *atpA* transcript ends

According to the overlapping model, one would expect that the 3′ *termini* of *atpH* mRNAs ending nearby a protein footprint (at position +297) overlap with the 5′ ends of transcripts encoded by the downstream gene, *atpF*. When primers were designed inside the coding region (f2 and f3 primers, Figure 1A), a sharp RT-PCR product was obtained with both TAP untreated and treated RNA samples (Figure 1B, lanes 10 and 11), indicating that the transcripts result from a processing event at the 5′end. As the cRT-PCR indicated only one mRNA type we directly sequenced the PCR product. We found that the *atpF* mRNAs displayed a 5′ end at position −45 from the *atpH* AUG and a 3′end in the inter-genic region between the *atpF* and *atpA* ORFs, at position +44 downstream to the *atpF* stop codon (f in Figure 3A and bottom panel). These data indicate that the *atpF* mRNAs are mostly di-cistronic (together with the *atpH* cistron) and contain very stable 5′ and 3′ ends. Therefore, we could not detect mono-cistronic *atpF* mRNAs with 5′-ends overlapping with the *atpH* 3′-ends. The existence of *atpH/F* transcripts was further confirmed by Northern hybridization, that revealed a very abundant band (f) of around 1300 nucleotide in size using specific probes hybridizing to the *atpF* coding region (Figure 2A and B, probe 5) and also to the *atpH* 3′ UTR (probes 2, 3, 4). No smaller bands corresponding to *atpF* mono-cistronic transcripts could be observed. We could not find the transcripts ending at position −30 upstream to the *atpF* ORF that were previously identified by primer extension [7], probably because they are very rare. In any case, no overlapping is possible between the 3′ ends of the *atpH* mRNAs ending at +297 (corresponding to position −166 upstream to the *atpF* start codon) and the 5′ ends of the *atpF* mRNAs starting at −30 (see Figure 3A).

On the other hand, a sRNA reflecting the footprint of a RNA-binding protein was identified at the 3′ end of the *atpH/F* co-transcripts at position +44 (corresponding to position −27 from the *atpA* AUG) [4]. To determine if the overlapping model applies to transcripts of the *atpF/A* adjacent genes, we next mapped the *atpA* mRNA ends. A weak and fuzzy PCR product was obtained (Figure 1B, lanes 12 and 13) when using the a1 and a3 primers (Figure 1A). We found that out of 8 clones, 5 clones presented the 5′ end at position −237 from the *atpA* start codon, inside the *atpF* coding region. The remaining 3 clones (3/8) presented the 5′ end at the −52 site from the *atpA* start codon, just upstream to the protein footprint, as expected by the overlapping model (Figure 3A, bottom panel). Both the −237 and the −52 ends result from a 5′ processing event as suggested by the presence of the amplified products also in the TAP-untreated sample (Figure 1B, compare lanes 12 and 13). However, surprisingly, all the clones presented truncated 3′ ends inside the *atpA* coding sequence (h and h′ in Figure 3A). Thus, we conclude that we could detect *atpA* mono-cistronic mRNAs with 5′ ends overlapping with *atpF* 3′ ends (Figure 3A, bottom panel). However, these transcripts are all truncated at their 3′ ends and cannot be translated into functional protein products. Other short *atpA* mRNAs initiating at position −74 from the AUG were previously mapped by RACE but were detected only at a very low level in primer extension experiments [7]. Concordantly, when a Northern blot hybridization was performed using a double stranded DNA probe hybridizing to the *atpA* coding sequence (Figure 2A and B, probe 6), no bands of the expected size for mono-cistronic *atpA* transcripts (around 1500 nucleotides) could be detected. Only longer RNAs of around 3500 nucleotides were clearly revealed (band g). Detection of the same RNAs (g) with probes 2–6 indicates that they correspond to poly-cistronic *atpH/F/A* mRNAs. The presence of abundant poly-cistronic *atpA* mRNAs is also supported by our previous analyses using the primer extension technique [7].

To map both ends of the poly-cistronic *atpH/F/A* mRNAs, we designed primers inside the coding sequences of the *atpH* (h2) and *atpA* (a2) genes (Figure 1A). We obtained a very sharp PCR band that could be directly sequenced (Figure 1B, lanes 14 and 15). The 5′ end of the *atpH/F/A* mRNAs maps at positions −45 upstream to the *atpH* AUG (Figure 3A, bottom panel) and the 3′ end maps at position +448 downstream to the *atpA* stop codon (g in Figure 3A). No sRNAs were found close to the +448 position [3]. However, the upstream 50 nucleotides can fold in a very stable stem loop structure with a predicted ΔG of −29.49 kcal/mol (using the MFOLD 3.2 program, http://mobyle.pasteur.fr/cgi-bin/portal.py?#forms) (Figure 3B, right panel). These data suggest that the 3′ end of the tri-cistronic *atpH/F/A* mRNAs is stabilized by a secondary structure rather than by a protein barrier.

Finally, we analyzed the *atpI/H/F/A* co-transcripts. Long transcripts of the expected size (around 4600 nucleotides) could be detected in Northern blot analyses exclusively with the *atpI* probe (Figure 2A and B, probe 1, star). These data suggest that the amount of poly-cistronic mRNAs covering the entire large *atp* operon (from *atpI* to *atpA* genes) is probably very small in relation to the total amount of different *atp* mRNAs. Concordantly, no PCR product was amplified when the primers were designed inside the *atpI* (i2) and the *atpA* (a2) coding regions (Figure 1A).

### Mapping of the primary *atpI/H* and *atpH* transcript ends

Finally, to determine if primary and 5′ processed transcripts differ at their 3′ ends, we mapped the *termini* of primary transcripts which are initiated from both the *atpI* and the *atpH* promoters. We first characterized the *atpI/H* co-transcripts, which are initiated at the position -229 from the *atpI* promoter [7]. We designed a reverse primer, i4 (Figure 1A), that was specifically hybridizing to the region between the transcription start site (−229) and the −48 processing site. This allowed to exclude all processed mRNAs and to increase the chance to clone the primary transcripts. As expected for primary transcripts, PCR amplification with i4 and h3 primers was obtained when RNAs were TAP-treated (Figure 1C, compare lane 1 with 2). All the clones that have been analyzed (11) contained 5′ ends around position −229. 7 of the 11 clones contained 3′ ends at position +151, where a stem-loop structure is predicted to fold. However, the remaining 4 clones had different 3′ ends, which were scattered within and in between the reading frames of *atpH* and *atpF* mRNAs (b in Figure 3A).

Furthermore, we characterized the primary *atpH* transcripts which are initiated at the −418 position from the internal *atpH* promoter [7]. We designed a reverse primer, h4 (Figure 1A), that was specifically hybridizing to the region between the internal transcription start site (−418) and the −45 processing site. As expected for primary transcripts, PCR amplification with primers h4 and h3 was obtained when RNAs were TAP-treated (Figure 1C, compare lane 3 with 4). A smear of PCR products was obtained indicating heterogeneous distribution of mRNAs. All the clones that have been analyzed (10) contained 5′ ends at position −418 but had different 3′ ends, which were scattered within and in between the reading frames of *atpH* and *atpF* mRNAs (c in Figure 3A). Altogether, these data demonstrated that a large part of the primary *atpI/H* and *atpH* transcripts is not stabilized at the 3′ end but is rapidly degraded. Degradation is more pronounced for the *atpH* mRNAs, but it is still detectable for the primary *atpI/H* mRNAs. Our results indicate that the primary and the processed mRNAs have different 3′ends and different stabilities, suggesting that a processing event at the 5′ ends might be required for definition and stabilization of the 3′ends.

## Discussion

Our study demonstrates that the 5′ ends of processed *atpI* and *atpH* mRNAs and the 3′ ends of processed *atpH* (at position +297) and *atpH/F* mRNAs map close to the sRNAs sites (Figure 3A), which were identified as footprints of RNA-binding proteins [3], [4]. Our data therefore support the model that RNA-binding proteins (PPR proteins or other classes of proteins with the same barrier function) are responsible for the protection of the mRNA ends from nuclease degradation. However, only a small proportion of *atpI* transcripts display overlapping ends with the *atpH* mRNAs, which are encoded by an adjacent cistron. In addition, the *atpA* RNAs, which map close to a protein footprint and overlap with the *atpF* transcripts of the upstream cistron, are truncated at the 3′ end and clearly correspond to degradation products. Therefore, the overlapping protection mechanisms appear to have only a limited role in obtaining stable processed mRNAs of the large *atp* operon in *Arabidopsis*. Based on our data we propose a new model for end definition of processed transcripts of the *atp* operon in *Arabidopsis* chloroplasts. The first site for RNA-binding proteins that follows a transcription initiation site serves to protect and define the 5′ end of the nascent poly-cistronic transcripts, while all the following protein-binding sites or hairpin structures that are encountered by the progressing RNA polymerase serve to define the 3′ ends of the transcripts. If this model applies also to other operons requires further investigations.

We observe that the processed *atpI/H* and *atpH* mRNAs are more efficiently protected at the 3′ end than the corresponding primary transcripts. Our data suggest that a processing action at the 5′ *terminus* might be required for definition and stabilization of the 3′ *terminus*, indicating a sort of communication between the ends of an RNA molecule during processing. Interestingly, our observations are reminiscent of the end-protection mechanisms of most eukaryotic nuclear transcripts where the CAP binding protein, protecting the 5′ ends, is deposed on the transcripts by the RNA polymerase II in a co-transcriptional manner [12] and enhances the processing events (cleavage and poly-adenylation) at the 3′ ends [13]. If also in chloroplasts the RNA-binding proteins are placed on the mRNAs by the RNA polymerase in a co-transcriptional manner is still an open question.

Our study indicates that both mechanisms of mRNA protection, either mediated by RNA-binding proteins or by hairpin structures, can operate in parallel and within the same operon. In addition, the mono-cistronic *atpH* mRNAs, which are by far the most abundant transcripts of the *atp* operon [10], are likely stabilized by a RNA-binding protein at the 5′ end and both a hairpin and a RNA-binding protein at the 3′ end. In case that the 3′>5′ exonuclease overruns the protein-mediated protection site, the degradation process can be then blocked by a second barrier, the hairpin structure. Thus in addition to transcription from an internal promoter and protein-mediated protection at the 5′ end, the amount of *atpH* mRNAs might be enhanced by a double protection at the 3′ end.

## Methods

### Plant growth conditions

*Arabidopsis* (*Arabidopsis thaliana*) seeds (ecotype Columbia) of wild-type plants were surface-sterilized for *in vitro* culture. Seeds were spread on MS agar plates with sucrose, kept for 72 h at 4°C in darkness and then grown at 23°C under a 16 h/8 h light/dark cycle at 70 µmol of photons m^−2^ s^−1^. Plants were harvested after 7 days.

### RNA purification

Frozen material of plants was ground in a mortar and re-suspended in 3 volumes of solution A (10 mM Tris-HCl pH8; 100 mM NaCl; 1 mM EDTA; 1% SDS) and 2 volumes of phenol/chloroform/isoamyl alcohol (25∶24∶1; v/v/v). After centrifugation, RNAs in the aqueous phase were again extracted twice with phenol-chloroform and finally once with chloroform. After over-night precipitation in 2 M LiCl at 4°C, RNAs were then precipitated in ethanol.

### Circular RT-PCR

In order to distinguish between primary and secondary transcripts, the first choice RLM-RACE kit was used (Ambion). In short, RNAs were first incubated with TAP (Tobacco acid pyro-phosphatase) and then self-ligated. 400 ng of RNAs were retro-transcribed using a reverse gene-specific primer and the SuperScript II enzyme (Invitrogen). The PCR reaction was performed in presence of forward and reverse gene-specific primers. PCR fragments were cloned into pCR2.1- TOPO vector (Invitrogen) and several clones were sequenced using a commercial service (Eurofins). In parallel, reactions without TAP treatment, reverse transcription or DNA template in the PCR amplification were carried out as controls.

Gene specific primers for retro-transcription were as follows:

for processed *atpI* and primary and processed *atpI/H* transcripts: i1 primer (5′-ctggaaaccccctatttgcc-3′);
for *atpH* primary and processed transcripts: h1 primer (5′-gtccaatagaagcaagc-3′)
for *atpH/F/A* transcripts : h1 primer (5′-gtccaatagaagcaagc-3′)
for *atpF* transcripts: f1 primer 5′-tcaatacaccgaaaactacac-3′;
for *atpA* transcripts: a1 primer (5′-ggtaccggtatttacaatcg-3′);

The reverse and the forward gene-specific primers for the PCR were as follows:

for processed *atpI* transcripts: i2 primer (5′-catattgccctctgacag-3′) and i3 primer (5′-gtgagtctatggaaggtc-3′)
for primary *atpI/H* transcripts: i4 primer (5′-gttttggatcccaactaaacaaatcac -3′) and h3 primer (5′-tcaaggtacagctgcggg-3′)
for processed *atpI/H* transcripts: i2 primer (5′-catattgccctctgacag-3′) and h3 primer (5′-tcaaggtacagctgcggg-3′)
for primary *atpH* transcripts: h4 primer (5′-tgatagtagttcctatcc-3′) and h3 primer (5′-tcaaggtacagctgcggg-3′)
for processed *atpH* transcripts: h2 primer (5′-caacagccaacccagcagc-3′)and h3 primer (5′-tcaaggtacagctgcggg-3′)
for *atpF* transcripts: f2 primer (5′-ggtattaaatccgaaactccc-3′) and f3 primer (5′-atcaagtccgcgaacggg-3′)
for *atpA* transcripts: a1 primer (5′-ggtaccggtatttacaatcg-3′) and a3 primer (5′-caacgattgcgtgagttactg-3′)
for *atpH/F/A* transcripts: h2 primer (5′-caacagccaacccagcagc-3′) and a2 primer (5′-gctctcaattaggtga-3′).

### Northern blot analysis

For Northern blot hybridization of *atpI* and *atpH* transcripts, gene-specific complementary primers were DIG-labeled with the DIG oligonucleotide tailing kit 2nd generation (Roche Diagnostics) and used as probe. RNAs (3 µg) were separated on denaturing formaldehyde (6%) agarose (1.3%) gels. RNAs were then blotted onto nylon Hybond-N+ membranes (Amersham Pharmacia) and hybridized to the DIG-labeled probe overnight at 65°C. Signal detection was performed using the DIG luminescent detection kit (Roche Diagnostics). *atpI* transcripts were detected using the primer 1 (5′-ctaaagcaaccgtcgtatttatatcattcgttggtgctgctaactccccttgaggtaac-3′); atpH transcripts were detected using the primers 2 (5′-gtccttcccaaggattgttgtctcaatgaataattgtaggagttaaatcttgatagaa-3′), 3 (5′-tttttaattttcaataataataatgagacttattagaattaagctagaatttgagaccaag-3′) and 4 (5′- cctatttggatatttgtaaacagaatcaaaaacctattctatttacaaacgtattttccaaa-3′) that hybridize to the *atpH-atpF* intergenic region.

For northern blot hybridization of *atpF* and *atpA* transcripts, PCR fragments have been 32P-labelled by random priming. Pre-hybridization (1 h at 65°C) and hybridization (over-night at 65°C) were performed in 0.5 M NaHPO4, pH 7. 2, 1 mM EDTA, 7% SDS and 1% BSA. After hybridization, filters were washed in 40 mM NaHPO4 at pH 7. 2, 1 mM EDTA and 7% SDS at room temperature for 10 min followed by washing at 65°C for 5 min. The *atpF* probe was amplified using the *atpF* coding DNA sequence (CDS) 5′ primer (5′-tcactggccatccgccg-3′) and *atpF* CDS 3′ primer (5′-cgtttctacgttacgcaagc-3′); the *atpA* probe was amplified using the *atpA* CDS 5′ primer (5′-ggtaaccattagagccgacg-3′) and *atpA* CDS 3′ primer (5′-gagcttaatttagcggctc-3′).
