# Correction: Complex Processing Patterns of mRNAs of the Large ATP Synthase Operon in *Arabidopsis* Chloroplasts

**DOI:** 10.1371/annotation/26e11ce5-3dc1-4f77-85b8-ded1849605e0

**Published:** 2013-12-11

**Authors:** Mustafa Malik Ghulam, Florence Courtois, Silva Lerbs-Mache, Livia Merendino

The name of the first author was incorrectly represented in the Citation. The correct Citation is: Malik Ghulam M, Courtois F, Lerbs-Mache S, Merendino L (2013) Complex Processing Patterns of mRNAs of the Large ATP Synthase Operon in Arabidopsis Chloroplasts. PLoS ONE 8(11): e78265. doi:10.1371/journal.pone.0078265 

